# Measuring Success: Evaluation Article Types for the *Public Health Education and Promotion* Section of *Frontiers in Public Health*

**DOI:** 10.3389/fpubh.2014.00111

**Published:** 2014-08-13

**Authors:** Matthew Lee Smith, Marcia G. Ory

**Affiliations:** ^1^Department of Health Promotion and Behavior, College of Public Health, The University of Georgia, Athens, GA, USA; ^2^Department of Health Promotion and Community Health Sciences, School of Public Health, Texas A&M Health Science Center, College Station, TX, USA

**Keywords:** evaluation, review criteria, public health education and promotion, article type, peer review

The aims of this article are to provide a rationale about the importance of evaluation in public health initiatives; justify *Public Health Education and Promotion*’s decision to create an Evaluation Article Type; and outline the evaluation criteria from which submitted articles will be assessed for publication.

## The Importance and Use of Evaluation in Public Health Education and Promotion

Evaluation is a process used by researchers, practitioners, and educators to assess the value of a given program, project, or policy (1). The primary purposes of evaluation in public health education and promotion are to: (1) determine the effectiveness of a given intervention and/or (2) assess and improve the quality of the intervention. Through evaluation, we can identify our level of success in evoking desired outcomes and accomplishing desired objectives. This is accomplished by carefully formulating specific, measurable objective statements that enable evaluators to assess if the intervention influences intended indicators and/or if the correct measures were used to gage effectiveness. Determining the impact of our efforts has vast implications for the future of the intervention. For example, through evaluation we are able to identify the essential elements of a given intervention (e.g., activities, content, resources, and structure), refine content and implement strategies, and decide whether or not to invest more resources for scalability.

High-quality evaluation is contingent upon the appropriateness of the design and selected measures for the questions being posed and the population being studied. Measurement is especially critical to evaluation because it enables the evaluator to know if changes or improvements occur as a result of the intervention, and it provides testable evidence for participant progress and program success. Evaluation is a critical factor for demonstrating accountability to all stakeholders included in the intervention. More specifically, conducting an appropriate and rigorous evaluation shows that the evaluator is accountable to the audiences and communities they serve, the organization for which they work, the funding agency supporting the project, and the greater field of public health.

Evaluation serves many varied purposes in addition to providing accountability for the stakeholders. At the very core, evaluative efforts help determine if predetermined objectives related to behavior change or health improvement were achieved in the proposed health education or promotion initiative. Evaluation is also useful to improve elements surrounding program implementation (e.g., partnership development, fidelity, effectiveness, and efficiency) and can increase the level of community support for a given intervention or initiative. Further, evaluation contributes to our knowledge about the determinants of health issues as well as the best and most appropriate public health interventions to address them. This knowledge is extremely valuable to guide future research and practice. Evaluation also informs policy decisions at the organizational, local, state, national, and international level.

## Evaluation Types

The role of evaluation has evolved over time. There are many types of evaluation, which are primarily defined by their design and purpose (2). The selection of an evaluation design is dependent upon the initiative’s focus, health issue being targeted, audience, setting, and timeline. Efficacy research includes evaluation performed under strict and regulated conditions, often in the form of randomized controlled trials (RCT). This type of evaluation is beneficial to determine what types of interventions work, while controlling for confounders and external influences. Effectiveness research includes evaluation performed in less controlled situations. This type of evaluation is beneficial to determine if the effects from RCT can be replicated in ‘real-world’ settings and conditions, often on a grander scale. Dissemination and implementation research typically includes evaluations performed in ‘real-world’ settings. This type of evaluation is beneficial to determine how to get what is known to be effective into the hands of the people, organizations, and communities that need them most. Much of this translational and pragmatic research includes evaluation about participant recruitment and retention, organizational adoption, fidelity, partnership formation and collaboration, data collection processes, scalability, and sustainability.

There are many phases of evaluation, which are primarily defined by their purpose and timing in the initiative’s delivery (3–5). *Formative evaluation* typically occurs in the early stages of an initiative to ‘pilot test’ for the purposes of obtaining feedback from involved parties, adjusting and enhancing the intervention components and content, and guiding the future directions of the initiative. Formative evaluation is most often concerned with feasibility and the appropriateness of materials and procedures. Formative evaluation permits preliminary testing and refinement of study hypotheses, data collection instruments, and statistical/analytical procedures. Generally, this form of evaluation occurs on a small scale to ensure unanticipated problems (e.g., glitches, breakdowns, lengthy delays, and departures from the design) are identified and the intervention quality is improved before ‘going to scale’ (i.e., prior to allocating larger investments of time, effort, and resources). *Process evaluation* is a type of formative evaluation that focuses on the intervention itself (as opposed to the outcomes) and should occur throughout the ‘life’ of an initiative. This type of evaluation uses data to assess the delivery of services and examine the nature and quality of processes and procedures. Process evaluation helps the evaluator to define the content, activities, and parameters of the initiative. It also addresses whether or not the intervention reached the intended audience, was appropriate for the audience, and was delivered as intended (including elements of fidelity and receipt of adequate intervention dose). *Summative evaluation* encompasses the overall merit of the intervention in terms of immediate impact as well as intermediate- and long-term outcomes. In addition to the intervention’s effectiveness, this type of evaluation also encompasses process evaluation, considering that predicted outcomes and objectives can only be achieved if the intervention is delivered with fidelity, as intended.

## Rationale for Introducing Evaluation Article Type Submissions

Recognizing the importance of evaluation, the *Public Health Education and Promotion* section has created an Article Type dedicated to evaluation. Evaluation is a special niche of public health education and promotion that assesses interventions’ ability to change health-related knowledge, perceptions, behavior, and service/resource utilization. While many public health education and promotion evaluations examine program efficacy and effectiveness, the emergent emphasis on translational issues of program dissemination and implementation (e.g., participant and delivery site recruitment and retention, fidelity, and maintenance/sustainability) requires the application of pragmatic research principles and methodologies (6, 7). Such translational evaluations address different research questions than traditional efficacy and effectiveness evaluations and are often conducted under pragmatic research designs. Pragmatic designs also attempt to promote the translation between research and practice (8). Thus, articles written using these methodological techniques require tailored review criteria to determine their appropriateness for publication.

Further, in public health, there are many types of innovations (e.g., trainings, courses, curricula, health promotion programs, and environmental or policy change), and there are many ways to report the participants, procedures, and findings of these initiatives based on the data collection methodology and research design (e.g., CONSORT for reporting controlled randomized trials, TREND for non-randomized evaluations, and STROBE for observational studies) (9–11). Although these guidelines are good for documenting the quality of the evaluation in terms of the appropriateness, sophistication, and replicability of the research design and evaluation, they are not all encompassing for innovations in public health education and promotion. As such, general, expansive, and all-encompassing set of criteria are needed to assess evaluation-related manuscripts submitted to the *Public Health Education and Promotion* section to ensure published manuscripts are rigorous, timely, relevant, and responsive to public health needs.

## Evaluation Article Type

*Public Health Education and Promotion* will accept a broad spectrum of articles that evaluate programs, courses, curricula, teaching methods, and other pedagogical elements as well as public health innovations at the organizational, environmental, or policy levels relevant to our mission. Such translational research articles will require a sufficient description of the program logistics, procedures, and participants/sample. Additionally, submissions will require a Discussion section that shares practical implications, lessons learned for future applications of the program, and acknowledgment of any methodological constraints. Articles should not exceed 6,000 words and include a maximum of five tables/graphs. Details about the Evaluation Article Type can be found online (http://www.frontiersin.org/Public_Health_Education_and_Promotion/articletype). A detailed description of the criteria used by Review Editors during the peer-review process is available as a separate file. While this information is obviously beneficial for Review Editors, we hope it will be consulted by authors prior to submitting evaluation-related manuscripts to *the Public Health Education and Promotion* section.

## Conflict of Interest Statement

The authors declare that the research was conducted in the absence of any commercial or financial relationships that could be construed as a potential conflict of interest.

## Evaluation Article Type Criteria for Peer Review *Public Health Education and Promotion*

Indicate what this article evaluates

__an educational or training program or intervention
__a course
__a curriculum
__a teaching method
__multiple pedagogical facets
__a health promotion program
__environmental, technological or policy natural or planned change
__none of the above (i.e., inappropriately categorized for submission as an Evaluation article)
__other. Please specify: ________________________________________________

Indicate the target audience

__community professionals (of one or more disciplines)
__healthcare professionals (of one or more disciplines)
__lay public
__other

Note: In the following questions, “intervention/program” is used to encompass the innovation (subject of the targeted effort, whether it is a course, curriculum, pilot project, program, etc.) being evaluated.

Ratings:

Significance of issue being addressed by the intervention/program: *(scored out of a maximum of 10 points)*
Appropriateness of the methodology used to address the question being asked: *(scored out of a maximum of 10 points)*
Quality of the writing: *(scored out of a maximum of 10 points)*
Quality of the figure(s) and tables: *(scored out of a maximum of 10 points)*
Significance of the evaluation findings: *(scored out of a maximum of 10 points)*
Application of the evaluation findings: *(scored out of a maximum of 10 points)*
Could this program be replicated by other organizations?
__Yes
__No
__Unclear based on the presented information

Mandatory Sections:

- An Evaluation article has the following mandatory sections: abstract, introduction, background and rationale, methods, results, discussion, and conclusions. Are all sections present?

Abstract:

- Is the abstract written in a clear and comprehensive way?

Introduction:

- Does the introduction present the problem in an appropriate context?
- Does the Introduction identify a knowledge gap justifying the need for evaluation?
- Other comments on introduction.

Background and Rationale:

- Is the literature review sufficient?
- Is the intent of the intervention/program adequately described?
- Are the questions asked by the evaluation those that are most essential to the success of the intervention/program being attempted?
- Other comments on background and rationale.

Methods:

- Is the population adequately described so it is clear: (1) how they were recruited? and (2) if they are representative of the broader population?
- Is the intervention/program adequately described (e.g., development, previous findings, components, format/design)?
- Are the intervention/program implementation procedures adequately described (e.g., structure, participant and site recruitment, data collection)?
- Is the evaluation methodology appropriate to assess the ***process?***
- Is the evaluation methodology appropriate to assess the ***outcome?***
- Are the statistical analysis techniques appropriate? Adequately presented?
- Other comments on methods.

Results:

- Are findings accurately reported from data presented?
- Is the level of detail of the results appropriate (too much, too little, about right)?
- Is essential information missing?
- Are effect sizes for outcomes available to enable cross-study comparison, when appropriate?
- Other comments on results.

Discussion:

- Are the reported findings summarized briefly and described within the context of what is currently known about the innovation (using findings from previous evaluations)?
- Does the discussion address the knowledge gap identified in the Introduction section?
- Does the discussion address all possible concerns of both internal and external validity of the findings (Limitations section)?
- Does the article conclude with practical recommendations for others who might replicate this intervention/program (or similar programs)?
- Does the article conclude with applied recommendations for practitioners in the field who might deliver this intervention/program (or similar programs) in their communities/settings?
- Does the evaluation contribute concrete recommendations for delivering and/or improving the intervention/program in future applications (directed toward researchers or practitioners, as appropriate)?
- Other comments on discussion.

Conclusion:

- Are the conclusions justified?
- Overall, does the article contribute to building Evidence-Based Practice?

References:

- Is prior work properly and fully cited?

Article Length:

- An Evaluation article should not exceed 4,500 words. Should any part of the article be shortened? If yes, please specify which part should be shortened.
- An Evaluation article should not include more than 5 tables/figures. If there are more tables/figures included, please specify if you believe tables can be combined, condensed, or eliminated.

Language and Grammar:

- Is the language and grammar of sufficient quality?
- Should the paper be sent to an expert in English language and scientific writing?

Other Comments:

- Please add any further comments you have regarding this manuscript.
